# Are antibacterial effects of non-antibiotic drugs random or purposeful because of a common evolutionary origin of bacterial and mammalian targets?

**DOI:** 10.1007/s15010-020-01547-9

**Published:** 2020-12-15

**Authors:** Axel Dalhoff

**Affiliations:** Institute for Infection Medicine, Christian-Albrechts-University of Kiel, Brunswiker Str. 4, 24105 Kiel, Germany

**Keywords:** Common ancestry, Conserved targets, Reversion of resistance, Virulence attenuation, Synergy

## Abstract

**Purpose:**

Advances in structural biology, genetics, bioinformatics, etc. resulted in the availability of an enormous pool of information enabling the analysis of the ancestry of pro- and eukaryotic genes and proteins.

**Methods:**

This review summarizes findings of structural and/or functional homologies of pro- and eukaryotic enzymes catalysing analogous biological reactions because of their highly conserved active centres so that non-antibiotics interacted with bacterial targets.

**Results:**

Protease inhibitors such as staurosporine or camostat inhibited bacterial serine/threonine or serine/tyrosine protein kinases, serine/threonine phosphatases, and serine/threonine kinases, to which penicillin-binding-proteins are linked, so that these drugs synergized with β-lactams, reverted aminoglycoside-resistance and attenuated bacterial virulence. Calcium antagonists such as nitrendipine or verapamil blocked not only prokaryotic ion channels but interacted with negatively charged bacterial cell membranes thus disrupting membrane energetics and inducing membrane stress response resulting in inhibition of P-glycoprotein such as bacterial pumps thus improving anti-mycobacterial activities of rifampicin, tetracycline, fluoroquinolones, bedaquilin and imipenem-activity against *Acinetobacter* spp. Ciclosporine and tacrolimus attenuated bacterial virulence. ACE-inhibitors like captopril interacted with metallo-β-lactamases thus reverting carbapenem-resistance; prokaryotic carbonic anhydrases were inhibited as well resulting in growth impairment. In general, non-antibiotics exerted weak antibacterial activities on their own but synergized with antibiotics, and/or reverted resistance and/or attenuated virulence.

**Conclusions:**

Data summarized in this review support the theory that prokaryotic proteins represent targets for non-antibiotics because of a common evolutionary origin of bacterial- and mammalian targets resulting in highly conserved active centres of both, pro- and eukaryotic proteins with which the non-antibiotics interact and exert antibacterial actions.

## Introduction

Two lines of evidence have demonstrated that non-antibiotics exert antibacterial activities. First, antibacterial effects of non-antibiotic drugs are well documented [1–9]. Agents discussed in comprehensive reviews will not be alluded to once again in this manuscript. Second, commonly used medications were found to have a significant impact on the faecal microbiome [9–11]. Proton pump inhibitors (PPIs), metformin used for the treatment of type II diabetes, laxatives, and psychotropic drugs had the biggest impact on the faecal flora; opioids, serotonine re-uptake inhibitors, tricyclic antidepressants, antihistamines, and even local anesthetics affected the faecal flora, too, but had a much smaller effect. The antibacterial effect of proton pump inhibitors is due to their proton dependent conversion into sulfen-amide derivatives with sulfides constituting the main end products of degradation. PPIs exhibit a non-selective antibacterial activity at an acidic environment but inhibit selectively *Helicobacter* spp*.* and *Campylobacter* spp. at a neutral pH. PPIs exert no specific antibacterial action since binding to a particular target was not observed. PPIs bind to a broad range of intracellular proteins in *Helicobacter* spp.; binding was enhanced in an acidic environment. The lipophilic nature of PPIs may also result in an unspecific interaction with cell membrane constituents [12–16]. The concentration- and time-dependent antihyperglycaemic effects of metformin are due to AMP-activated protein kinase (AMPK) dependent and independent effects including their downstream effects. Furthermore, metformin inhibited mitochondrial respiration and probably also mitochondrial glycerophosphate dehydrogenase [17, 18]. Copper complexation may also contribute to the antihyperglycaemic action of metformin [19, 20]. Metformin exerted effects not only on the gut microbiome but also against *Legionella pneumophila* [21] and *H. pylori* [22–24] and several other bacterial- and viral species [25] in vitro, in vivo and in the clinical setting. Proposed mechanisms of antibacterial action encompass those effects attributed to antihyperglycaemic effects of metformin, inhibition of electron transport, and also AMPK independent effects such as immunomodulation, or production of mitochondrial reactive oxygen species thus enhancing bactericidal activities of macrophages [25, 26]. Laxatives constitute a diverse group of agents inhibiting multiple targets with a wide range of biological effects including anti-bacterial-, -viral-, -fungal-, -inflammatory-, and -oxidant activities [27]. Other laxatives bind irreversibly to nucleophilic amino acids, thus inactivating unspecifically the corresponding proteins [28, 29]. Psychotropic drugs interacted with bacterial membranes and transport systems [5, 9]. These data demonstrate that those non-antibiotic drugs exerting most frequently pronounced antibacterial activities affect bacteria unspecifically and target multiple functions.

However, non-antibiotics may hypothetically interact specifically with prokaryotic targets because of structural and/or functional homologies of eukaryotic- and prokaryotic proteins. Evidence has been provided in a companion paper [30] that antibiotics interact with eukaryotic targets because of evolutionarily conserved functions. This review summarizes data describing antibacterial activities of non-antibiotics with processes considered to be essential in pro—as well as eukaryotes. These essential reactions may be evolutionary highly conserved thus offering the chance for inhibitory activities of drugs beyond the borders of the domains eukarya, archae and bacteria. Selected examples for evolutionarily conserved processes are first, phosphorylation and dephosphorylation as a frequently employed mechanism of signalling; second, initiation and propagation of electrical signalling by voltage-gated ion channels; third, metalloenzymes with zinc as a key ion for catalytic functions and as an essential structural element.

## Phosphorylation and dephosphorylation

Reversible phosphorylation and dephosphorylation of proteins are essential for the regulation of protein activity and signalling in pro- and eukaryotes. Protein phosphorylation triggers essential processes like cell wall biosynthesis in bacteria and neurologic- or immune responses, endocrine actions, etc. in humans [31–33]. Therefore, any interaction with protein phosphorylation or dephosphorylation has far-reaching ramifications.

### Inhibition of bacterial serine/threonine/tyrosine protein kinases

Posttranscriptional modification of eukaryotic proteins is primarily accomplished by protein phosphorylation of serine, threonine or tyrosine catalysed by “Hanks-type kinases”, i.e. serine/threonine and sometimes also serine/tyrosine protein kinases (STPKs). Bacterial STPKs were defined as “eukaryotic-type kinases”, but comprehensive phylostratigraphic analysis suggests that Hanks-type kinases present in eu-, prokaryotes and archaea all share a common evolutionary origin [34]. In general, the catalytic regions of bacterial, animal, and human STPKs and phosphorylases show strong homologies [35–44]. Penicillin-binding proteins (PBPs), being serine transferases [45], and STPKs are linked to an ancillary domain named penicillin-binding and serine/threonine kinase-associated (PASTA). An extracellular PBP domain characterizes PASTA proteins and an intracellular STPK domain that is similar to those of acyl serine transferases found in mammals [46]. PASTAs regulate bacterial metabolism, cell division, and cell wall homeostasis through the recognition of muropeptides and also sense and respond to hostile environmental stress factors such as an immune response or limited nutrient supply as well as antibiotic stress. Therefore, PASTA proteins play a central role in cell wall biosynthesis, virulence and β-lactam resistance [47–50]. PASTA domains are found in both, the C-terminus of PBPs as well as in bacterial STPKs [51–55]. While the STPK domain is well conserved, PASTA domains are quite divergent [56]. STPKs are involved in antibiotic-resistance as it influences the expression of low-affinity class B PBP5, PBP2X, and PBP2a being associated with cephalosporin-resistance in *E. faecium* [57–59], as well as penicillin-resistance in *S. pneumoniae* [60–63], and methicillin-resistance in *S. aureus* [64–67], respectively. Likewise, STPKs play a role during growth and β-lactam susceptibility of *Corynebacterium* spp. [68, 69], *B. subtilis* [70–74], *M. tuberculosis* [60, 75–80], and other species. Therefore it is not unexpected that several kinase inhibitors of diverse structural classes used in human medicine increased susceptibilities of various bacterial species to cell wall active antibacterial agents.

In general, PASTA kinase mutants are hypersusceptible to β-lactams and inhibition of kinases sensitizes in particular Gram-positive bacteria and also Gram-negative bacteria to β-lactams. Antibacterial activities of most of the agents were examined by monitoring time-kill curves so that minimal inhibitory concentrations (MICs) have rarely been reported. Screening of a natural compound library of low molecular weight kinase inhibitors revealed that most of the compounds inhibited in combination with a sub-MIC concentration of nafcillin growth of the methicillin-resistant *S. aureus* (MRSA) test strain. Staurosporine, an alkaloid isolated from *Streptomyces staurosporeus* exhibiting unspecific anti-cancer activity due to activation of caspase thus inducing apoptosis, was used as a comparator. The effect of staurosporine on β-lactam susceptibilities was however not due to caspase activation but attributable to a selective inhibition of PASTA kinases. Staurosporine caused in combination with a sub-MIC concentration of nafcillin a 68% growth inhibition, whereas several staurosporine derivatives inhibited the growth of the MRSA test stain to 100% [81, 82]. Staurosporine and the cyclin-dependent kinase inhibitor, AZD5438, sensitized *L. monocytogenes* to ampicillin, ceftriaxone, cephalexin, and lysostaphin 10- to 100-fold, whereas the activity of vancomycin was not enhanced by staurosporine [83]. Both, inhibition of the kinase activity and sensitization of *L. monocytogenes* to β-lactams were concentration dependent and parallel processes. An imidazopyridine aminofurazan (GSK690693) and a pyrazolopyridazine-derivative (GW779439X) sensitized *L. monocytogenes* [84] and MRSA [85, 86], respectively, to various β-lactams (Table 1) via inhibition of STPKs. Camostat, an inhibitor of transmembrane protease serine 2 (TMPRSS2), commercially available in Japan for treatment of chronic pancreatitis and postoperative reflux esophagitis, was found to inhibit viruses such as SARS-CoV 2 [87, 88] and Gram-positive bacteria. MICs of camostat and its derivatives gabexate and nafamostat ranged from ≥ 500 to 50 μM after an 18–20 h incubation in the absence of β-lactams. However, nafamostat exhibited a bactericidal activity during a 6 h incubation period reducing the inoculum by four log_10_ titres [89, 90].

**Table 1:** Antibiotic susceptibilities of *L. monocytogenes* and methicillin-resistant *S. aureus* to various β-lactams in the absence or presence of PASTA kinase inhibitors GSK690693 and GSK690693, respectively (modified according to 81, 82; n.d. = not done)

Agent	Wild type		PrkA PASTA kinase mutant		Wild type		Stk1 PASTA kinase mutant	
	Without	With	Without	With	Without	With	Without	With
	20 μM GSK690693				5 μM GSK690693			
	*L. monocytogenes*				Methicillin resistant *S. aureus*			
Ampicillin	0.25	0.06	0.031	0.031	n.d.	n.d.	n.d.	n.d.
Ceftriaxone	8.0	1.0	0.062	0.062	32	16	16	16
Oxacillin	n.d.	n.d.	n.d.	n.d.	16	1.0	1.0	1.0
Ceftaroline	n.d.	n.d.	n.d.	n.d.	1.0	0.5	0.5	0.5
Meropenem	0.25	0.125	0.031	0.062	0.25	0.125	0.125	0.125
Nafcillin	n.d.	n.d.	n.d.	n.d.	16	2.0	2.0	2.0
Vancomycin	8.0	8.0	8.0	8.0	1.0	1.0	1.0	1.0

Imidazopyridine aminofurazans (IPA) inhibited the kinase PknB of mycobacteria and potentiated activities of β-lactams against *Mycobacterium* spp. Incubation of M*. smegmatis* and *M. chelonae* with MIC_50_-concentrations of meropenem in combination with various IPAs resulted in an inhibition of the test strains by 2.0–0.5 and 8.0 mg/L meropenem, respectively as compared to MICs of 4.0 and 16 mg/L in the absence of IPAs. Likewise, incubation of *M. abscessus* to a fixed MIC_50_-concentration of ampicillin in combination with various IPAs resulted in an inhibition of the test strain by 12.5 mg/L as compared to a MIC of 125 mg/L in the absence of IPAs. The inhibitors potentiated activities of normally ineffective β-lactams against *Nocardia* spp. [91, 92]. Aminobenzimidazoles sensitized *M. smegmatis* and *M. tuberculosis* to 14 β-lactams reducing their MICs from ≥ 256 to 1 to 16 mg/L, except for cephalothin, cefadroxil, carbenicillin and piperacillin [93]. Other aminobenzimidazoles sensitized MRSA, methicillin-resistant *S. epidermidis,* and multidrug-resistant *P. aeruginosa* as well as *A. baumanii* not only to penicillin G, oxacillin, and methicillin but also to novobiocin, colistin, tobramycin, ciprofloxacin lowering MICs up to 512-fold [94–96]. Another aminobenzimidazole suppressed carbapenem-resistance in NDM-1 producing strains of *K. pneumoniae* [97]. Yet another aminobenzimidazole inhibited a histidine kinase two-component signalling system. It reconstituted colistin activity in multidrug-resistant *A. baumannii* and *K. pneumoniae* by down regulating the *pmrCAB* operon thus reverting phosphoethanolamine modification of lipid A which causes colistin resistance [98].

The tricyclic antihistamine loratadine inhibited biofilm formation and PASTA kinases Stk and Stk1 in *S. epidermidis* and *S. aureus*, respectively, resulting in increased activities of β-lactam antibiotics against MRSA and increased activities of both, β-lactams and vancomycin in vancomycin-resistant strains of *S. aureus* [3, 99]. A small-molecule quinazoline compound (Inh2-B1) inhibited specifically Stk1 in *S. aureus.* It reduced MBCs of ceftriaxone and cefotaxime for MRSA from ≥ 100 to ≤ 4 mg/L [100]. The finding that loratadine resensitized MRSA to vancomycin [99] is in contrast to previous reports [83–85] that STPK inhibitors revert activities of β-lactams only but not other antibiotic classes. This contradiction may be resolved by the finding that another STPK, i.e. Stp1, contributes to reduced vancomycin susceptibilities in *S. aureus* as this kinase regulates virulence and cell wall thickness [101, 102].

Several groups synthesized agents derived from protein kinase inhibitor pharmacophores with intrinsic antibacterial activities. An unsaturated crotonic acid derivative (IMB-YH-8) exerted an anti-mycobacterial activity in itself due to STPK inhibition [103]. This molecule inhibited selectively the kinases PknA and PknB of *M. tuberculosis*. PknB modulates the SigH regulatory pathways, which regulate a transcriptional network responding to various stresses. The MICs of IMB-YH-8 for *M. tuberculosis* ranged from 0.25 to 1.0 mg/L irrespective of whether the test strains were susceptible or mono- or multiply resistant to isoniazid, rifampin, streptomycin, ethambutol and ofloxacin [103]. The anthraquinone derivative mitoxantrone used in cancer therapy was found to inhibit PknB in *M. tuberculosis*, thereby preventing its growth [104]. Plant-derived trypsin inhibitors were active against Gram-positive as well as Gram-negative bacteria and exhibited anti-viral activities [105].

AMPKs are highly conserved in yeast, plants, and mammals. It is assumed that AMPKs evolved in the early eukaryote to control the output of carbohydrates produced by acquired bacterial endosymbionts that developed into mitochondria but were thought not to play a role in bacteria [106–108]. However, AMPKs were isolated from *L. pneumophila* [21], *H. pylori* [22–24], *Mycobacterium* spp., *B. subtilis*, *L. monocytogenes*, and rhizobial bacteria [109–112] and were involved in sensitization of resistant bacteria to antibiotics, too [10, 70, 83–85]. Bacterial AMPKs may be inhibited by metformin.

Antibiotic phosphorylating kinases confer resistance to aminoglycosides, macrolides, as well as phenicols and are organized in the antibiotic kinome consisting of the antibiotic resistome and the microbial kinome [113, 114]. It has been shown that the aminoglycoside modifying enzymes *O*-phosphotransferases are structurally and functionally ortholog to eukaryotic STPKs [115–119]. Pro- and eukaryotic kinases, which are most likely to be simultaneously inhibited by a common ligand, were identified based on the similarity in their ligand-binding profiles rather than via their sequence similarity. A survey of 150,000 kinase inhibitory values, comprising more than 3800 compounds tested against a panel 172 kinases revealed that pyrazolo-pyrimidines had nanomolar affinity against many STPKs involved in cancerogenesis [120] and two of them (pyrazolo-pyrimidines) as well as the flavone quercetin inhibited bacterial *O*-phosphotransferases and reverted aminoglycoside resistance [121–123]. Quercitin occupied the ATP binding site and formed several hydrogen bonds with the phosphotransferase APH(2″)-IVa. Furthermore, flavonoids such as quercitin chelate with metal cations thus inhibiting aminoglycoside-acetyltransferase activity and sensitizing aminoglycoside-resistant strains to these agents [124]. Apart from inhibition of aminoglycoside-phosphotransferases and aminoglycoside-acetyltransferases flavonoids interact unspecifically with bacterial membranes and efflux pumps, so that they exert antibacterial activities in themselves [27, 125] and exert additive or synergistic combination effects in particular with cell-wall active agents [125]. Several STPK substrates were phosphorylated by *O*-phosphotransferases and vice versa, STPK inhibitors such as several isoquinoline-sulfonamides or the casein kinase-1 inhibitor CKI-7 inhibited *O*-phosphotransferases. Unfortunately, these inhibitors were active in a cell-free system only but did not reverse aminoglycoside-resistance in living bacteria [126, 127]. The phosphoinositide 3-kinases inhibitor wortmanin inhibited APH(2″)-Ia because of structural homologies of lipid kinases and aminoglycoside modifying enzymes [128].

These data demonstrate that pro- and eukaryotic STPKs share common ancestors and exhibit significant structural and functional homology. Therefore, non-antibiotic inhibitors of eukaryotic STPKs inhibited specifically just one or two bacterial STPKs thus reverting antibiotic resistance in Gram-positive as well as Gram-negative bacteria. It is an open question why the inhibitors just interact with specific bacterial STPKs, e.g. with mycobacterial PknA and PknB out of 11 STPKs, two tyrosine-protein phosphatases and one serine/threonine protein phosphatase, and leave the others unaffected. However, this phenomenon may offer the chance to synthesize inhibitors of structurally and functionally related targets but being sufficiently different to allow for selectivity for pro- but not eukaryotic STPKs and in parallel inhibiting specifically just the relevant bacterial STPK.

### Disabling bacterial pathogenesis by targeting host cell serine/tyrosine kinases

Enteropathogenic *E. coli* (EPEC) attach to epithelial cells and express several virulence factors such as the bacterial outer membrane protein intimin which mimics a ligand-receptor interaction. One essential factor causing adherence of EPEC to host intestinal epithelial cells is “translocated intimin receptor” (Tir) which spans the host plasma membrane and binds intimin on the bacterial surface to the epithelial cell resulting in tight adherence of the bacterium to the host cell and formation of lesions. These lesions are characterized by a loss of intestinal microvilli and the formation of actin-filled membranous pedestals that protrude beneath the adherent bacterium. Phosphorylation of Tir by multiple host cell tyrosine kinases, in particular members of the Abl family of tyrosine kinases, is crucial for the formation of actin pedestals and its inhibition or deletion results in a loss of virulence [129, 130]. Pyrido-pyrimidine compounds are developed to treat cancers caused by dysregulated Abl and were found to inhibit bacterial Abl family tyrosine kinases thus blocking pedestal formation and consequently virulence [130].

In general, Abl family tyrosine kinases play an important role in the pathogenesis of e.g. *S. flexneri*, *H. pylori*, *S. enterica*, *P. aeruginosa*, *C. trachomatis*, *M. tuberculosis*, *Anaplasma phagocytophilum*, and also viruses and parasites. A recent, comprehensive summary [131] has presented data demonstrating that Abl family tyrosine kinases phosphorylate microbial factors required for pathogen entry into-, release from-, and/or motility within host cells. Imatinib and other FDA-approved ATP-competitive inhibitors of Abl family tyrosine kinases affected host cell cytoskeletal dynamics required for cellular protrusions being essential for adhesion of bacteria to and release from host cells thus reducing the virulence of the pathogens.

### Inhibition of bacterial serine/threonine phosphatases

As phosphorylation of STPKs is stable partner serine/threonine phosphatases are needed to reverse the regulation. Recently, a serine/threonine phosphatase has been identified in *E. coli* and has been characterized biochemically [132]. It shows significant homologies to human phosphatase 2C (PP2C) phosphatases [35, 133–136], so that bacterial homologs are referred to as eukaryote like serine/threonine phosphatases (eSTPs). It is well documented that inhibitors of human phophatases cyclosporin A, tacrolimus (FK506), and rapamycin exhibit anti-fungal and viral but no anti-bacterial activities. However, highly conserved FK506 binding proteins (FKBPs) exist in *Legionella* spp., *Chlamydia* spp., *N. meningitidis*, and *P. aeruginosa* [137]. FKBPs of these bacterial species function as outer membrane virulence factors. These virulence factors may possibly be inhibitable by cyclosporin A, tacrolimus, and rapamycin as these phosphatase inhibitors bind to FKBPs with high affinity. Tacrolimus and rapamycin inhibited intracellular survival of *Legionella* spp*.* theoretically via inhibition of virulence [138] thus indicating that inhibitors of human serine/threonine phosphatases may attenuate bacterial virulence. This finding was probably not followed up consistently as it is generally believed that cyclosporin A, tacrolimus, and rapamycin are not antibacterially active.

## Calcium channel blockers

Voltage-dependent calcium channels (Cavs) couple membrane energetics with Ca^2+^-signalling thereby regulating essential physiological processes. Cavs are highly conserved and are phylogenetically related to bacterial voltage-gated sodium channels (BacNaVs). Previously it was thought that bacteria lack Cavs [139–144]. A bacterial sodium channel from *Bacillus halodurans* was characterized. Commercially available calcium channel blockers inhibited this NaChBac channel. The dose-response curves for dihydropyridines were comparable to those for mammalian ion channels [140]. Furthermore, first prokaryotic calcium channels were identified recently. The newly characterized two BacNav homologs, CavMr from *Meiothermus ruber* and NavPp from *Plesiocystis pacifica*, are selective for Ca^2+^, and selective for Na^+^ with Ca^2+^-dependent inhibition, respectively [145]. The amino acid sequences of mammalian Cavs and bacterial BacNavs are very similar and the quaternary structures and functional determinants of BacNavs are well defined [146–150] so that they are used as a model for gating and ion permeation.

It has been described that some calcium channel blockers exhibit quite low in vitro antibacterial activities in the absence of antibiotics with MICs ranging from 10 to 200 mg/L [151–157] (Table 2). Verapamil was tested against *M. tuberculosis* and *M. abscessus* only and was inactive [156, 157]. However, verapamil exhibited bactericidal activity against *P. aeruginosa* at a concentration of 0.98 μg/mL although its MIC value was as high as 12,768 μg/mL [158]. Also, lacidipine and nifedipine were bactericidally active at twice their MICs and reduced inocula of *V. cholera* and *S. aureus* [152] or *Shigella *spp*.* and *S. typhimurium* [153], respectively, by 5–8 log_10_ titres within 12 h. Likewise, verapamil, nifedipine, nisoldipine, and nitrendipine inhibited *E. coli* chemotaxis in the micromolar range. At concentrations around tenfold higher than that needed for inhibition of chemotaxis, each of these antagonists inhibited motility. While ≥ 1 mM of verapamil did not reduce viable counts of *E. coli*, 5 μM each of nifedipine, nisoldipine, and nimodipine reduced survival of the test strain to 50% within 30 min [159]. Verapamil, nitrendipine, and nifedipine reduced spore germination in *B. megaterium* at a concentration of 1 mM each [160]. Thus, calcium channel blockers affected bacterial physiology at relatively low concentrations and exhibited even a bactericidal activity during the 30 min and 12 h incubation period, respectively, without exhibiting a relevant—if any—effect on the discrete endpoint MIC. Concentrations of calcium antagonists exhibiting in vitro activities against Gram-positive and Gram-negative bacteria should be related to serum concentrations following single oral doses as specified in Table 2. This comparison indicates that even the lowest MICs exceed mean maximal serum concentrations significantly by more than one order of magnitude. However, verapamil, nitrendipine, nisoldipin, nimodipine, nifedipine, and lacidipine affected physiological functions and even viability of the test strains at low concentrations. Similarly, lactividine, amlodipine and verapamil showed protective effects in vivo. Concentrations achieved in experimental animals were significantly lower than their MICs as the agents were administered at human-equivalent and thus subinhibitory doses [152–155, 161, 162]. Lactividine protected animals from death in a mouse model of *V. cholera* infection; it reduced viable counts dose dependently and inhibited cholera toxin production in vivo [152, 154]. Amlodipine acted as a tissue protectant in experimentally induced *S. aureus* rhinosinusitis. Both, monotherapy with cefazolin or amlodipine reduced numbers of macrophages in epithelial cells by 57% and 39%, respectively [155]. Coadministration of verapamil with sub-inhibitory doses of bedaquiline achieved an equivalent anti-mycobacterial effect as the full dose of bedaquilin. This adjunctive effect of verapamil may permit the administration of lower doses of bedaquilin [156, 157].

**Table 2:** Minimal inhibitory concentrations (mg/L) of calcium channel blockers (modified according to 152–158) (^a^MIC for a single test strain; otherwise, ranges and concentration inhibiting 50% of the strains studied (MIC_50_) are provided; p*K*_a_ = p*K*_a_ is the negative log_10_ of the acid dissociation constant *K*_a_ of a solution; log*P* = octanol/water distribution coefficient (p*K*_a_ and log*P* values are quoted from DrugBank); n.t. = not tested)

	Benidipin, felodipin, nitrendipine, nimodipin	Lacidipin	Nifedipin	Amlodipin	Verapamil
Physicochemistry					
p*K*a	5.41–7.89	6.4	5.33	9.45	9.68
log*P*	3.21–4.36	5.18	2.49	2.22	5.23
Pharmacokinetics	Single oral standard dose, mean maximal serum concentration following oral administration				
		4 mg = 3.5 μg/L	10 mg = 18 μg/L	10 mg = 5.9 μg/L	120 mg = 219 μg/L
Bacterial species	Minimal inhibitory concentrations, range or ^a^MIC_50,_ mg/L				
*Bacillus* spp.	≥ 800	10–> 200, 25	25^a^	25–200, 50	n.t.
* S. typhimurium*	≥ 800	n.t.	n.t.	50^a^	n.t.
* S. typhi*	≥ 800	n.t.	25^a^	50^a^	n.t.
* S. aureus*	≥ 800	10–200, 25	25^a^	10–400, 25	n.t.
* E. coli*	≥ 800	50–> 200, 100	> 200	> 800^a^	n.t.
* Klebsiella* spp.	≥ 800	50–> 200, 200	50^a^	400–800	n.t.
* Hafnia* spp.	≥ 800	50^a^	n.t.	n.t.	n.t.
* Proteus* spp.	≥ 800	200–≥ 400, 200	n.t.	n.t.	n.t.
* Providencia* spp.	≥ 800	100^a^	n.t.	n.t.	n.t.
* Citrobacter* spp.	≥ 800	> 200^a^	n.t.	n.t.	n.t.
* P. aeruginosa*	≥ 800	25–≥ 200, 50	> 200	> 800	n.t.
* P. putida*	≥ 800	50–100, 50	n.t.	n.t.	n.t.
* Pasteurella septica*	≥ 800	10^a^	n.t.	n.t.	n.t.
* V. cholerae*	≥ 800	10–200, 25	25^a^	25^a^	n.t.
* V. parahaemolyticus*	≥ 800	10–200, 25	n.t.	n.t.	n.t.
* Shigella* spp.	≥ 800	10–≥ 200, 50	10–50, 25	25–200	n.t.
* M. tuberculosis*	n.t.	n.t.	n.t.	n.t.	≥ 512
* M. abscessus*	n.t.	n.t.	n.t.	n.t.	≥ 250

Calcium antagonists improved in vitro activities of tetracycline and fluoroquinolones against *S. aureus, Enterobacteriaceae* and non-fermenters irrespective of whether the strains were tetracycline- or fluoroquinolone-resistant [158–160]. It also improved activities of ofloxacin, rifampicin, and bedaquilin, but not isoniazid or amikacin against *M. tuberculosis* as well as *M. abscessus* (Table 3) [161–169]. Verapamil also increased the intraphagocytic activities of isoniazid and rifampicin [168] as well as bedaquilin and moxifloxacin [168] against drug-susceptible and drug-resistant *M. tuberculosis* and also the intracellular activity of azithromycin against *L. monocytogenes* [170, 171] and that of daptomycin against *S. aureus* [172]. Resistance phenotypes but not resistance mechanisms were described for these test strains so that it may be probable but it is not proven that antibiotic resistances were caused by efflux mechanisms which were inhibited by calcium channel blockers. Likewise, amlodipine enhanced the activities of imipenem against multidrug-resistant *A. baumanii* by inhibiting expression of the resistance nodulation cell division efflux pump AdeABC [173].

Verapamil is assumed to be an inhibitor of P-glycoprotein and efflux pumps in *M. tuberculosis* and other bacterial species, so that its potentiating activity was attributed to an intraphagocytic and/or intrabacterial drug accumulation [157, 169–177] and also increased bioavailability due to interaction with CYP3A4 [157, 178]. Interestingly, the ABC transporter isolated from *Lactococcis lactis*, LmrA, exports intracellular amphiphilic compounds. When LmrA was expressed in human lung fibroblasts it substituted for P-glycoprotein and conferred multidrug resistance on these human cells. This effect was due to almost identical biochemical characteristics of LmrA and P-glycoprotein showing the same substrate affinities. The activities of both were equally well affected by verapamil [179–182]. Homologs of LmrA have been found in a variety of bacterial pathogens, suggesting that this resistance mechanism is ancient and plays a crucial role in procaryotes and that its functional homolog, the P-glycoprotein, mediates drug resistance in eukaryotes [183]. In agreement with this theory is the finding that *E. coli* strains lacking the AcrAB pump are hypersusceptible to calcium channel blockers suggesting that verapamil and other calcium channel blockers are substrates of this bacterial pump. Furthermore, subinhibitory concentrations of verapamil abolished in *E. coli* the proton motive force, decreased intracellular ATP concentrations, and reduced the growth rate without affecting the synthesis of DNA, RNA and proteins in general or RecA protein in particular. This finding excludes the possibility that cell division could have been inhibited due to an induction of the SOS response. However, verapamil may likely have perturbed the integrity of the bacterial membrane and may have disrupted the assembly of the FtsZ complex [184]. In addition, verapamil affected transport activity of the OpuA (Glycine-betaine transporter) protein from *Lactococcus lactis* reconstituted into membrane vesicles [183], due to its accumulation in the inner leaflet of the vesicle. These data not only support the theory that extrusion of drugs represents an ancient resistance mechanism with homologous P-glycoproteins structures in pro- and eukaryotes with which calcium channel blockers interact but also suggest that in addition calcium channel blockers affect membrane functions [185, 186].

The theory of an augmented activity of antibiotics due on inhibition of efflux pumps by calcium-channel blockers in both, pro- and eukaryotes, resulting in increased intracellular concentrations has been questioned. The alternative model is based on the physicochemical characteristics of verapamil with a p*K*_a_ value of 9.68 and a log*P* value of 5.23. Therefore, verapamil is a lipophilic weak base with a protonated tertiary amine group at neutral pH. At a pH of almost 10 verapamil exists in its least ionized state, so that it has a high affinity to neutral lipid bilayers. At a neutral pH and in particular at a pathophysiologically relevant slightly acidic pH, however, verapamil is positively charged, so that it adheres with high affinity to and inserts into negatively charged lipid membranes thus disrupting membrane energetics and inducing membrane stress response [187, 188]. This interaction with membranes due to physicochemical mechanisms explains why verapamil reduced viable counts of *M. tuberculosis* by 8 log_10_ titres within 15 h although bacteria did not replicate and were deprived of nutrients. In addition, membrane disruption was not due to increased intramycobacterial drug accumulation [188]. These data suggest that augmentation of antibiotic activities was not caused by direct inhibition of efflux pumps by verapamil resulting in drug accumulation, but rather by dissipation of the proton motive force, which in turn affects most efflux pumps so that drug accumulation was an indirect consequence of perturbed membrane energetics resulting in altered efflux pump function. In agreement with this conclusion, it was shown that incorporation of verapamil [188, 189] and dihydropyridines [190–194] into the cell- and model membranes affected P-glycoprotein activity. In general, modulation of membrane functions due to physicochemical interactions of dugs with bacterial- and mammalian membranes, respectively, are prevalent amongst almost every antibiotic class provided the agents interact as catamphiphilic drugs with membranes [195, 196]. Physicochemical constants summarized in Table 2 indicate that lipophilicity and ionization vary considerably amongst the calcium channel blockers. Therefore, the P-glycoprotein inhibitory potential of the most lipophilic calcium antagonist verapamil is more pronounced than that of less lipohilic dihydropyridines (e.g. IC_50_-values of nitrendipine and verapamil, respectively, were 18.2 μM versus 2.8 μM for digoxin transport); analogous data were obtained for other dihydropyridines as compared to verapamil [197–200]. These data demonstrate that all the calcium-antagonists tested interacted primarily with membranes before drug binding to L-type calcium channel receptors thus secondarily affecting P-glycoprotein functions [187, 188, 193]. It has to be considered that ionization of agents depends on environmental pH values which vary according to pathophysiologically relevant conditions [196]. Furthermore, the pH gradient in microbes varies significantly with the environmental pH, so that also the membrane potential varies with external pH [197–202]. Thus, membrane disruption is mutually dependent from the ionization of the agent and the environmental pH, so that the real level of inhibition is a function of the specific condition at the focus of infection and may be variable and hardly predictable.

Calcium channel blockers exert multiple actions in pro- and eukaryotes. In particular, they augmented antibacterial activity and reversed antibiotic resistance in Gram-positive and Gram-negative bacteria*.* Thus, calcium channel blockers could be used as an adjunct to antibacterial therapy. Antibiotic-calcium channel blocker combinations may result in a beneficial additive or even synergistic antibacterial effect and/or resistance reversal provided dose regimens are optimally adjusted.

The discrepancies between conclusions to be drawn on the basis of the static endpoint parameter MIC demonstrating that calcium channel blockers are devoid of a relevant in vitro antibacterial activity and their pronounced in vitro bactericidal activity and interference with cellular functions as well as their beneficial in vivo efficacy in experimental animals give rise to the question if MIC testing is an appropriate method to analyse antibacterial effects of non-antibiotics. These doubts are reasonable as bacterial voltage-gated calcium flux is correlated with physiological processes like chemotaxis, synthesis of pathogenicity factors, sporulation and modulation of the transcriptome [41, 201, 202]. Therefore, endpoints for a phenotypic examination or functional analysis of antibacterial activities of non-antibiotic drugs should mirror both, the mode of action of and the physiological processes triggered by the non-antibiotic drug. Dynamic physiological processes cannot be described by the static endpoint MIC.

## Inhibitors of angiotensin-converting enzyme and other zinc-containing enzymes

The angiotensin I converting enzyme (ACE, or kininase II) is a bivalent zinc-dependent dipeptidyl carboxypeptidase catalysing the conversion of angiotensin I into angiotensin II. Displacement of zinc from the active site inactivates ACE [203–205]. The thiol- and carboxyl-group, respectively, of ACE inhibitors such as captopril, elanapril and lisinopril, bind directly to the catalytic zinc. Removal of the thiol-group of captopril or its replacement with a carboxylic acid led to complete loss of activity [206–208]. Metallo-β-lactamases (MBLs), too, are characterized by conserved zinc ion binding sites in their active centres. Therefore, it was hypothesized that ACE inhibitors could probably bind to MBLs. Several studies have confirmed that ACE inhibitors form complexes with MBLs [209–213] due to binding of their thiol groups to both active site Zn^2+^ ions of MBLs of various bacterial species (summarized in [209]). Furthermore, captopril has structural similarity to the MBL degraded penicillin, so that captopril bound to MBLs most similar to that of hydrolysed β-lactams [209]. Such functional homologies suggest that the active sites of MBLs and ACE, respectively, and the mode of binding to their active centres may represent conserved structures. The IC_50_ values of the most active D-stereoisomer of captopril for various MBLs ranged from 0.07 μM for VIM-2, 1.7 μM for VIM-4, 7.2 μM for IMP-1, 20.1 μM for NDM-1 up to 262.8 μM for SPM-1 [209, 210]. The high affinity of D-captopril to VIM-2 was due to additional interactions observed between VIM-2 and the carboxyl-group of captopril as well as due to additional hydrogen bonds between VIM-2 and other MBLs with captopril [209–214]. The combination effect of D-captopril with meropenem was tested against a panel of *E. coli*,* K. pneumoniae*,* S. marcescens*, and *P. aeruginosa* strains producing VIM-2, VIM-4, IMP-4 and NDM-1 β-lactamases or NDM-1 β-lactamase in combination with CTX-M or TEM-type β-lactamases. Meropenem MICs for these strains ranged from 1 to 512 mg/L as compared to < 0.125 mg/L for the wild type strain. D-captopril reduced the MICs of meropenem for the resistant strains by two- to four dilution steps [209] so that 8 out of 11 strains with MICs greater than the resistant breakpoint of 8 mg/L became susceptible. Using a disc diffusion test it was demonstrated that l-captopril inhibited the growth of *E. coli* and *S. enterica* at ≥ 25 mg/mL [215]. Ramipril inhibited *M. chelonae* at 1.3 mg/L and *M. abscessus* at ≥ 5.2 mg/L [216]. Some investigational captopril derivatives as well as some approved thiol-containing drugs such as thiorphan, dimercaprol, and tiopronin inhibited activities of NDM-1, VIM-1, and IMP-7 at concentrations lower than those of captopril [217–219].

Apart from the functional homology between captopril and β-lactams, ACE is an evolutionary highly conserved protein. DNA sequence analysis revealed that ACE-homologos could be identified in a variety of different eukaryotic phyla and in procaryotes [220–227]. Some of these ACE-like enzymes have been purified and biochemically characterized in vitro. The bacterial ACE-like enzymes from *E. coli* (EcDCP), *Xanthamonas citri* (XcACE), and dipeptidyl carboxypeptidases from *Pseudomonas* spp., *P. maltophilia*, *Corynebacterium equi*, *B. subtilis* and *B. pumilus* have retained their ability to hydrolyse angiotensin I, among other ACE substrates, and were inhibited by relevant ACE inhibitors. Sequence identities between the enzymes vary from 91 to 73% for XcACE and human N-ACE or ACE2, and 20–8% for EcDCP and the human enzymes, respectively [227–229]. Although the overall sequence similarity may be low in some cases, the active site is highly conserved, so that ACE-inhibitors inactivate the enzymes irrespective of their origin effectively. Regardless of the functional and genetic homologies between pro- and eukaryotic ACE homologs, the function of the prokaryotic ACE-like enzymes has still to be defined in most cases. A dipeptidyl-carboxypeptidase with defined activity has been isolated from *S. gordonii*. The enzyme was a functional homolog of human ACE and was eightfold more active than the recombinant human ACE. The bacterial enzyme was able to hydrolyse the alpha- and beta-chains of fibrinogen. However, 1 μM each of captopril, lisinopril and enalapril, did not inhibit the bacterial enzyme activity. This discrepancy may be due to the fact that human recombinant ACE is a zinc-dependent dipeptidyl-carboxy-peptidase while the bacterial enzyme also behaves as a dipeptidyl-carboxypeptidase, but without an absolute requirement for metal ions, as neither EDTA nor EGTA inhibited the activity of the bacterial enzyme. On the other hand, divalent metal ions caused a more than twofold increase in enzyme activity.

Carbonic anhydrases (classes α, β, and γ) are essential metalloenzymes which play crucial roles in the entire animate system. Their physiological roles, mechanisms of action and agents used clinically as carbonic anhydrase inhibitors have been described in comprehensive reviews [230–232]. The active site of these enzymes consists of Zn^2+^ with which metal complexing anions or sulfonamides like acetazolamide, methazolamide, ethoxzolamide, dichlorophenamide, dorzolamide and brinzolamide, used clinically in glaucoma patients to lower the intraocular pressure, interact [232, 233]. Carbonic anhydrases could be isolated from a large variety though not from all bacterial species [234] and were inhibited by the sulfonamides acetazolamide and methazolamide (summarized in [231]). Inhibition of carbonic anhydrases leads to growth impairment of bacteria, viruses, and yeasts, but species-specific sulfonamide derivatives have been synthesized (summarized in [233]). However, bacterial species being devoid of carbonic anhydrases were nevertheless inhibited by sulfonamides [235] probably due to their classical mode of action as a PABA antagonist. Clinical efficacy of acetazolamide 500 mg b.i.d. for 4 days was examined in a pilot study in eight volunteers with active *H. pylori* infection. The urea breath test reverted in none of the patients to negative [236] possibly due to an effect of acetazolamide on acid secretion, which may have prevented an effect on *H. pylori*. Thus, inhibition of bacterial carbonic anhydrases by sulfonamides may represent a third target for novel sulfonamides in addition to their antagonism with para-aminobenzoate and their function as alternate substrates for pteridine resulting in an inhibition of dihydropteroate synthase. Captopril and elanapril inhibited carbonic anhydrases, too, but not as effectively as sulfonamides [237].

## Efflux pump inhibitors and non-steroidal anti-inflammatory drugs in particular

Efflux pumps are highly conserved structures in pro-and eukaryotes. Comprehensive reviews have summarized a plethora of data describing interactions of a broad variety of agents with efflux pumps [238–244]. In general, non-steroidal anti-inflammatory drugs (NSAIDS) and acetylsalicylic acid (aspirin^®^), its major metabolite salicylic acid, and acetaminophen (paracetamol^®^) in particular, interact not only with efflux pumps but exert pleiotropic antibacterial activities against bacteria many of which are equivocal [245–249]. Notably, the effect of NSAIDS on bacterial efflux pumps was contradictory as growth in the presence of (acetyl-) salicylic acid either caused intrinsic multiple drug resistance and increased virulence or reduced resistance and virulence, so that growth in the presence of salicylate can be both beneficial and detrimental. The reader is kindly referred to the comprehensive review articles for more detailed information [245–249].

**Table 3:** Minimal inhibitory concentrations of ofloxacin (OFX), ciprofloxacin (CPX), rifampicin (RIF), amikacin (AMK), isoniazid (INH), tetracycline (TET), and bedaquiline (BDQ), alone and in combination with verapamil (n.t. = not tested; ^a^*n* = 6; ^b^*n* = 1; ^c^*n* = 19; modified according to 162–169)

Agent	*S. aureus*		*M. tuberculosis*		*M. abscessus* (*n* = 31)	
	Verapamil 100 μg/mL		Verapamil 128 μg/L		Verapamil 40 mg/L	
	Without	With	Without	With	Without	With
OFX	n.t.	n.t.	1.0–2.0^a^	1.0–0.5^a^	n.t.	n.t.
OFX	n.t.	n.t.	16^b^	16^b^	n.t.	n.t.
CPX	5.0 (*n* = 2)	< 0.25 (*n* = 2)	n.t.	n.t.	n.t.	n.t.
CPX	10–80 (*n* = 5)	< 2.5–5 (*n* = 5)	n.t.	n.t.	n.t.	n.t.
RIF	n.t.	n.t.	0.5–16^a^	0.125–0.5^a^	n.t.	n.t.
RIF	n.t.	n.t.	1024–2048^a^	16–512^a^	n.t.	n.t.
AMK	n.t.	n.t.	1.0–2.0^a^	0.25–0.5^a^	n.t.	n.t.
AMK	n.t.	n.t.	640^b^	640^b^	n.t.	n.t.
INH	n.t.	n.t.	5–10^a^	3–5^a^	n.t.	n.t.
INH	n.t.	n.t.	512^b^	512^b^	n.t.	n.t.
BDQ	n.t.	n.t.	0.03–1.0^c^	0.003–0.25	0.125–1.0	0.003–0.25
BDQ	n.t.	n.t.	4–8 (*n* = 5)	0.01–4 (*n* = 5)	n.t.	n.t.
TET	< 2.5 (*n* = 1)	< 2.5 (*n* = 1)	n.t.	n.t.	n.t.	n.t.
TET	10–80 (*n* = 12)	< 2.5–5 (*n* = 12)	n.t.	n.t.	n.t.	n.t.

## Conclusions and open questions

Data summarized above support the hypothesis that prokaryotic proteins may represent targets for non-antibiotics because of a common evolutionary origin of the corresponding pro- and eukaryotic proteins. The phylogenetically related and structurally highly conserved targets provide the rational basis for antibacterial actions of non-antibiotics, and vice versa interactions of antibiotics with mammalian targets [30]. Advances in structural biology, genetics, bioinformatics etc. resulted in the availability of an enormous pool of information enabling the analysis of the ancestry of pro- and eukaryotic genes and proteins. Comparisons of gene- or protein-sequences provide information about structural and/or functional convergencies or divergencies. Although pro- or eukaryotic enzymes may differ structurally hence being non-homologous they may be functionally homologous catalysing the same biological reactions because of their highly conserved active centres [250] as exemplified e.g. by eukaryotic ACE and the homologous enzymes in bacteria [227–229]. Bacterial—as well as mammalian active centres of STPKs, voltage-gated ion channels, ACE and MBLs are ancient and highly conserved so that agents interfering with activities of mammalian proteins inhibited the bacterial counterparts as well. Representative examples constituting key physiological reactions are summarized in Table 4. Protease inhibitors inhibited bacterial STPKs to which PBPs are linked [81–112] so that these drugs synergized with β-lactams and attenuated bacterial virulence. Calcium antagonists blocked not only pro- and eukaryotic ion channels [150–172] but interacted with negatively charged cell membranes thus disrupting membrane energetics and inducing membrane stress response [186, 187], so that P-glycoprotein like bacterial pumps were affected. ACE-inhibitors and sulfonamides interacted with zinc-metalloenzymes such as eukaryotic ACE and prokaryotic MBLs [209–219] or eu- and prokaryotic carbonic anhydrases [230–235].

**Table 4:** Effects of non-antibiotics on augmentation of antibacterial activity, reversal of resistance, and attenuation of virulence due to functional and/or structural homologies of human and bacterial targets, respectively (STPK = serine/tyrosine-protein kinase; PBP = penicillin-binding protein; MBL = metallo-β-lactamases

Bacterial receptor protein	Human target	Agents studied	Effect on bacterium
Phosphorylation and dephosphorylation			
Intracellular STPK linked to an extracellular PBP	Activation of caspase thus inducing apoptosis STPK	Staurosporine, investigational STPK-inhibitors, β-lactams or vancomycin	Synergy with β-lactams, but not vancomycin
Intracellular STPK linked to an extracellular PBP	Transmembrane protease serine 2 (TMPRSS2)	Camostat, nafamostat, gabexate	Antibacterial activity against Gram-positive bacteria
O-Phosphotransferase structurally similar to STPKs	Phosphatidylinositol 3-kinase (PI 3-kinase)	Investigational agents, wortmanin	Reversion of aminoglycoside resistance
Tyrosine kinases causing adherence to host cells	Tyrosine kinase inhibitor	Imatinib	Reduction of attachment to epithelial cells, thus attenuation of virulence
FK506-binding protein, a serine/threonine phosphatase	Calcineurin, a serine/threonine phosphatase	Ciclosporin, tacrolimus (FK506), rapamycin	Attenuation of virulence
Ion channel blocker			
Bacterial voltage gated ion channels	Calcium antagonists blocking L-type Ca-channels	Nitrendipine, nifedipine, nisoldipine, verapamil, lacidipine	Inhibition of motility, chemotaxis; attenuation of virulence; rapid and pronounced, but transient bactericidal activity
Bacterial voltage gated ion channels	Calcium antagonists blocking L-type Ca-channels	Verapamil in combination with rifampicin, tetracycline, fluoroquinolones, bedaquilin	Improved anti-mycobacterial activity
Ade ABC efflux pump	Calcium antagonists blocking L-type Ca-channels	Amlodipine in combination with imipenem	Enhanced activity of imipenem against *Acinetobacter baumanii*
Physicochemical interaction with bacterial membranes resulting in modification of membrane structure and function	Binding to and insertion insertion into membranes dependent on ionization of the agent	Verapamil	Subinhibitory concentrations of verapamil abolished in *E. coli* the proton motive force and decreased intracellular ATP concentrations
Inhibitors of zinc containing enzymes			
Metallo-β-lactamases	Angiotensin converting enzyme	Captopril, elanapril	Inhibition of various MBLs (e.g. VIM-2, VIM-4, IMP-1, NDM-1)
Carbonic anhydrases	Human carbonic anhydrases	Sulfonamide derivatives used for treatment of glaucoma	Growth impairment

These examples demonstrate that non-antibiotics most frequently support and augment antibacterial activities of antibiotics but exerted an antibacterial activity on their own in rare cases only. As none of the non-antibiotics discussed above interacted antagonistically with various antibiotics—except NSAIDs with frequently unpredictable synergistic or antagonistic interactions [245–249]—they could be administered in combination to improve antibacterial therapy and to reverse antibiotic resistance. Furthermore, the data summarized above could possibly provide models for target interactions with utility for future drug design. Screening could be based on the strategy to identify proteins of convergent evolution as these could represent essential targets.

Open questions are if protease-inhibitor and β-lactam hybrids could be synthesized targeting in parallel STPK—as well as PBP-domains of PASTA proteins?

Could Zn^2+^ chelating ACE-inhibitors and Zn^2+^ chelating antibiotics [30] mutually affect each other positively or negatively? Could a combination of non-antibiotics with antibiotics both carrying mitochondrial liabilities increase or decrease their individual effects on mitochondrial functions? Calcium antagonists [251–255] and ACE-inhibitors [256–260] reduced respiration and ATP synthesis. Sorafenib directly impaired mitochondrial function at clinically relevant concentrations [261] whereas imatinib lacked direct mitochondrial effects but affected mitochondrial functions via altered kinase and other signalling pathways [261, 262]. Likewise, ciclosporin A, rapamycin, and tacrolimus did not directly induce mitochondrial dysfunction but decreased energy production [263, 264]. Thus, almost all of the non-antibiotics mentioned above affect mitochondrial functions being either part of their modes of action or potential toxicities. Many antibiotics cause mitochondrial dysfunction, too, thus explaining their anti-neoplastic activities [30], or possibly promoting tumorigenesis [265–268], obesity [269], and psychiatric disorders [270, 271]. Thus, antibiotics as well as non-antibiotics exert beneficial or detrimental anti-mitochondrial activities. The question if combinations of non-antibiotics with antibiotics may increase or decrease drug inherent anti-mitochondrial effects of the respective drug thus affecting their efficacy and/or toxicity has not yet been assessed. It might be reasonably assumed that such drug combinations have been prescribed frequently to certain populations like cancer- or geriatric patients in whom polypharmacy is frequent but evidently having been without peculiarities so far [272].

Other open questions such as drug/drug interactions, impact on the microbiomes, etc. are beyond the scope of this review article but will need to be kept in focus. In summary, drug development could benefit from new perspectives on protein evolution. The currently available data permit an immediate implementation of well-justified combination therapies and may indicate perspectives for the future.

## Addendum search strategy

Publications addressing four topics were screened: first, phosphorylation and dephosphorylation with the keywords “Hanks-type kinases”, serine/threonine-, serine/tyrosine protein kinases (STPKs), serine/threonine phosphatases, penicillin-binding and serine/threonine kinase-associated (PASTA) protein, and the corresponding inhibitors. Second, initiation and propagation of electrical signalling by voltage-gated ion channels with the key words calcium antagonists, dihydropyridines, benzodiazepines, phenylalkylamines and the corresponding drugs, in particular nitrendipine and its derivatives, amlodipine, and verapamil. Third, angiotensin I converting enzyme (ACE, or kininase II) with the keywords ACE-inhibitors, in particular captopril. Fourth, evolutionary originin-, ancestry of prokaryotic, eukaryotic, archaeal proteins or active centres, homolog, ortholog, paralog. Additional keywords were antibiotics in general and sulfonamides, β-lactams, aminoglycosides, macrolides, chloramphenicol, oxazolidinones, tetracyclines, fluoroquinolones and the corresponding single agents of these drug-classes. Search strategy and selection criteria were based on the combination of keywords. Articles summarized in recent reviews were excluded from this synopsis and the reviews are quoted instead.
